# Social environment and genetics underlie body site‐specific microbiomes of Yellowstone National Park gray wolves (*Canis lupus*)

**DOI:** 10.1002/ece3.7767

**Published:** 2021-06-21

**Authors:** Alexandra L. DeCandia, Kira A. Cassidy, Daniel R. Stahler, Erin A. Stahler, Bridgett M. vonHoldt

**Affiliations:** ^1^ Ecology and Evolutionary Biology Princeton University Princeton NJ USA; ^2^ Smithsonian Conservation Biology Institute National Zoological Park Washington DC USA; ^3^ Yellowstone Center for Resources National Park Service Yellowstone National Park WY USA

**Keywords:** genetics, gray wolf, host–microbe interactions, mammal, microbiome, pedigree, social behavior, wild canid

## Abstract

The host‐associated microbiome is an important player in the ecology and evolution of species. Despite growing interest in the medical, veterinary, and conservation communities, there remain numerous questions about the primary factors underlying microbiota, particularly in wildlife. We bridged this knowledge gap by leveraging microbial, genetic, and observational data collected in a wild, pedigreed population of gray wolves (*Canis lupus*) inhabiting Yellowstone National Park. We characterized body site‐specific microbes across six haired and mucosal body sites (and two fecal samples) using 16S rRNA amplicon sequencing. At the phylum level, we found that the microbiome of gray wolves primarily consists of Actinobacteria, Bacteroidetes, Firmicutes, Fusobacteria, and Proteobacteria, consistent with previous studies within Mammalia and Canidae. At the genus level, we documented body site‐specific microbiota with functions relevant to microenvironment and local physiological processes. We additionally employed observational and RAD sequencing data to examine genetic, demographic, and environmental correlates of skin and gut microbiota. We surveyed individuals across several levels of pedigree relationships, generations, and social groups, and found that social environment (i.e., pack) and genetic relatedness were two primary factors associated with microbial community composition to differing degrees between body sites. We additionally reported body condition and coat color as secondary factors underlying gut and skin microbiomes, respectively. We concluded that gray wolf microbiota resemble similar host species, differ between body sites, and are shaped by numerous endogenous and exogenous factors. These results provide baseline information for this long‐term study population and yield important insights into the evolutionary history, ecology, and conservation of wild wolves and their associated microbes.

## Graphical abstract

We characterized the host‐associated microbiome across multiple body sites in gray wolves living in Yellowstone National Park, USA. Leveraging genetic, microbial, and observational data, we determined that social environment and genetics primarily underly microbial community composition in this wild mammalian system. Photo credit: NPS/Jacob W. Frank, Jim Peaco, and Daniel Stahler.

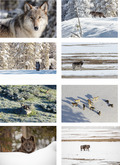

## 1 INTRODUCTION

Widespread interest in host‐associated microbiomes has led to critical insights about their form and function. Far from idle passengers, commensal microbes affect host development (Dominguez‐Bello et al., 2019), metabolism (Martin et al., 2019), immunity (Honda & Littman, 2012; Thaiss et al., 2016), reproduction (Al‐Nasiry et al., 2020), stress tolerance (Stothart et al., 2016), and behavior (Ezenwa et al., 2012), among other processes. Although originally limited to human and model systems, there has been a recent surge of studies characterizing microbiota in diverse host taxa. These studies traverse medical (Gupta et al., 2020), veterinary (Rodrigues Hoffmann et al., 2014), and conservation (Trevelline et al., 2019) communities, and include host systems ranging from invertebrates (Petersen & Osvatic, 2018) through humans (Peterson et al., 2009). Examples within Mammalia include carnivorans (Guo et al., 2018; He et al., 2018) cetaceans (Hooper et al., 2019; Sanders et al., 2015), chiropterans (Avena et al., 2016; Ingala et al., 2019), marsupials (Alfano et al., 2015; Cheng et al., 2015), primates (Clayton et al., 2016; Gomez et al., 2015), rodents (Lavrinienko et al., 2018; Suzuki et al., 2019), and ungulates (Gibson et al., 2019; Sun et al., 2019) sampled in captivity and the wild.

Despite increased study, there remain numerous questions about the primary factors underlying microbial species presence and abundance. Of particular interest is the contribution of host genetics, demography, and environment (Bonder et al., 2016; Ceja‐Navarro et al., 2015; Goodrich et al., 2014; Kurilshikov et al., 2017; Rothschild et al., 2018; Spor et al., 2011). As microbiota function in diverse physiological processes, elucidating these factors can have important implications for the evolutionary history, ecology, and conservation of species (DeCandia et al., 2018; Hauffe & Barelli, 2019; Trevelline et al., 2019).

Within wildlife systems, studies often consider broad‐scale patterns of phylosymbiosis or the eco‐evolutionary scenario where host phylogenetic relationships are mirrored by dissimilarity between host‐associated microbiomes (Brooks et al., 2016). This pattern has been observed within numerous host lineages, including invertebrates, rodents, and primates (Brooks et al., 2016). However, the taxonomic scale of comparison can strongly influence the degree of congruence between host phylogenies and microbial dendrograms, as these patterns break down within speciose host genera (Greene et al., 2019; Grond et al., 2020). Further, environmental and behavioral variables often explain significant portions of variance alongside phylogenetics. Metagenomic analyses of gut microbiomes across Mammalia, Aves, Reptilia, Osteichthyes, and others found evidence that host taxonomy, diet, lifespan, and behavior (i.e., activity and social structure) influenced microbial composition to varying degrees (Levin et al., 2021). Similar analyses of 16S rRNA across Mammalia, Aves, Reptilia, Amphibia, and Actinopterygii revealed that diet primarily predicted functional guilds and host phylogeny predicted the specific microbes present (Youngblut et al., 2019). The same pattern emerged within Mammalia, where diet was predictive of gut microbiome convergence at higher taxonomic rankings (such as microbial phylum) and host phylogeny was predictive of gut and skin microbial communities at lower taxonomic rankings (such as microbial family; Nishida & Ochman, 2018; Ross et al., 2018).

These broad‐scale studies provide valuable insights into the evolutionary history of hosts and their associated microbes. However, they lack details that may be relevant to the ecology and conservation of lower host clades. In order to obtain finer‐scale information, researchers have turned to species‐specific studies. Within captive management settings, microbiome analyses have yielded important information about the reproductive (Southern white rhinoceros, *Ceratotherium simum simum*; Williams et al., 2019) and gastrointestinal (red wolf, *Canis rufus*; Bragg et al., 2020) health of captive‐housed wildlife. Across host species, artificial diet and housing conditions significantly influence microbiota, as seen in primate microbes “humanized” by captivity (red‐shanked douc, *Pygathrix nemaeus,* and mantled howler monkey, *Alouatta palliata*; Clayton et al., 2016). While critical for ex situ conservation management, captive studies fail to capture wild microbes that colonize hosts in their natural habitat. Consequently, in situ studies are required to disentangle the evolutionary and ecological factors shaping wild microbiomes. These factors may include disease (Santa Catalina Island fox, *Urocyon littoralis catalinae*; DeCandia et al., 2020), habitat fragmentation (common vampire bat, *Desmodus rotundus*; Ingala et al., 2019) and geography, diet, and anthropogenic pressure (western lowland gorilla, *Gorilla gorilla*; Gomez et al., 2015), with increasing interest in the contribution of within‐species host genetics (house mouse, *Mus musculus*; Suzuki et al., 2019).

The majority of in situ microbiome studies focus on gut microbiota (Gomez et al., 2015; Ingala et al., 2019; Sanders et al., 2015; Suzuki et al., 2019), with a few studies targeting skin sites (Avena et al., 2016; Hooper et al., 2019). While informative, narrow focus on one or two sample types provides an incomplete characterization of commensal microbes. Body sites exhibit distinct microbial communities, due to differences in oxygen exposure, nutrient availability, substrate, and environmental factors (The Human Microbiome Project Consortium, 2012). For example, human guts harbor far more anaerobic species than human skin, due to low oxygen availability in the gastrointestinal tract (Coates et al., 2019). Further differences exist between skin microenvironments, where dry sites (such as the forearm) exhibit higher species richness than sebaceous sites (such as the forehead; Byrd et al., 2018; Grice & Segre, 2011). Similar patterns are observed in domestic dogs (*Canis familiaris*), where microbial species richness is highest at haired body sites (such as the axilla) and lowest at specialized mucosal sites (such as the nostril; Rodrigues Hoffmann et al., 2014). Considered together, this evidence suggests that different factors may influence microbiota at each body site, as seen in wild bank voles (*Myodes glareolus*; Lavrinienko et al., 2018). This necessitates study of body site‐specific microbiota in the wild to more holistically characterize host‐associated microbiomes.

**FIGURE 1:**
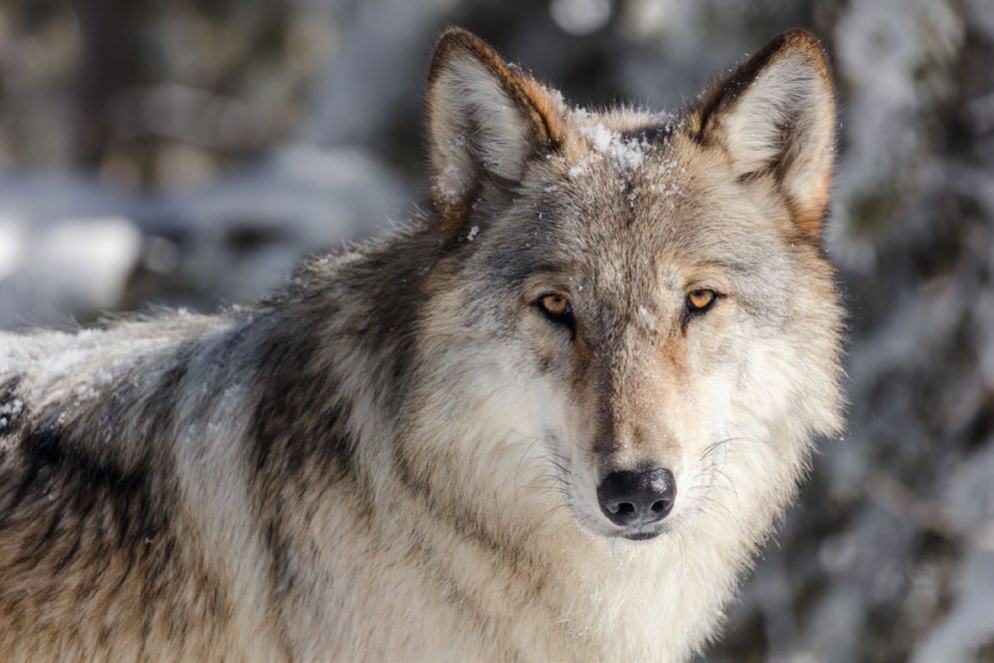
Gray wolves were reintroduced to Yellowstone National Park in 1995–1996 and have been closely monitored ever since. Photo Credit: NPS/Jacob W. Frank

The present study bridges these knowledge gaps by characterizing in situ wildlife microbiomes across multiple body sites. Specifically, we sequenced host‐associated bacterial communities across six haired and mucosal body sites (and two fecal samples) in a wild pedigreed population of gray wolves (*Canis lupus*) inhabiting Yellowstone National Park (YNP) in Wyoming, USA (Figure 1). We hypothesized that body sites would harbor distinct microbial communities, with haired sites exhibiting higher alpha diversity than mucosal sites, and fecal sites hosting higher proportions of anaerobic bacteria than nonfecal sites. We further leveraged observational data, biobanked blood and tissue, and the highly resolved YNP wolf pedigree (vonHoldt et al., 2020) to examine genetic, demographic, and environmental factors underlying body site‐specific microbiota in this wild mammalian system. Given that wolves live in kin‐structured family groups comprised of close relatives (Stahler et al., 2020; vonHoldt et al., 2008), we predicted that genetic relatedness would positively correlate with microbial similarity, as seen in studies of humans and model systems (Bonder et al., 2016; Spor et al., 2011). However, as many wolves disperse from their natal groups, we also predicted that unrelated wolves sharing the same social environment (here termed “sampling pack”) would exhibit more similar microbiota than unrelated wolves sampled in different packs, as seen in cohabitating humans (Dill‐McFarland et al., 2019; Song et al., 2013). Pack mates cooperatively share social contacts, resources, and prey items, including elk and (to a more variable degree) bison, deer, moose, or beaver (Metz et al., 2020)—it therefore follows that they share microbes, as well. The social structure of wolves, coupled with the availability of host genetic, microbial, demographic, environmental, and pedigree information provided the unique opportunity to examine body site‐specific microbiota and their underlying factors in a wild mammalian system. Results yielded system‐specific insights, while also contributing to the larger‐scale effort of characterizing wildlife microbiomes in situ.

## 2 MATERIALS AND METHODS

### 2.1 Sample and data collection

Gray wolves have been monitored annually by the National Park Service (NPS) since their reintroduction to YNP in 1995 and 1996. Static (e.g., sex) and dynamic (e.g., pack membership) life history variables were collected from aerial and ground surveys conducted during the winter monitoring season. In addition, biological samples were collected between December and February during helicopter captures (whole blood sampled through venipuncture) and field necropsies (tissue sampled from deceased individuals). At the time of microbiome sample collection, body condition was qualitatively scored by two handlers based on sex‐ and age‐specific patterns of weight, muscle/fat condition, coat condition, and injuries/illness detected. All capture and handling protocols were conducted in accordance with the NPS (IACUC permit IMR_YELL_Smith_wolves_2012) and Princeton University (Princeton IACUC #2009A‐17) Institutional Animal Care and Use Committees.

We generated genomic data using whole blood and microbiome data using skin swabs and fresh scat. We used sterile BD BBL™ CultureSwab™ swabs to sample commensal bacteria at six body sites, including ear canal, nostril, lip commissure, axilla, dorsal flank, and perianal area (Figure S1). At each body site, we rubbed the swab tip on the skin roughly 100 times, rotating by 90° every 25 times. We also collected fresh scat if the individual released feces during or immediately prior to sampling. Across three field seasons, we collected 151 microbiome samples from 25 unique individuals. We collected 56 samples from nine wolves during the 2017–2018 season, 35 samples from eight wolves during the 2018–2019 season, and 60 samples from 10 wolves during the 2019–2020 season. Two wolves were resampled in different field seasons, with wolf 1106M sampled in 2017–2018 and 2018–2019, and wolf 907F sampled in 2017–2018 and 2019–2020. One wolf (949M) was sampled after death following complications due to canine distemper virus (*Canine morbillivirus*). In total, we collected 25 ear canal swabs, 25 nostril swabs, 26 lip commissure swabs, 24 axilla swabs, 24 dorsal flank swabs, 25 perianal swabs, and 2 fresh scat samples. Upon collection, all samples were stored at −80°C until DNA extraction.

### 2.2 Genomic DNA extraction, RAD sequencing, and data analysis

We extracted DNA from whole blood following the Qiagen DNeasy Blood and Tissue Kit manufacturer protocol (Qiagen Inc.), quantified DNA using Quant‐iT™ PicoGreen™ dsDNA assays or high‐sensitivity Qubit™ fluorometry (Thermo Fisher Scientific), visualized extracts on 1% agarose gels to assess molecular weight, and standardized concentrations to 5 ng/μl. We then generated genomic data using a modified restriction site‐associated DNA sequencing protocol designed by (Ali et al., 2016) and described in (DeCandia et al., 2021; vonHoldt et al., 2020). Briefly, we digested DNA with the restriction enzyme *sbfI* and ligated uniquely barcoded, biotinylated adaptors. We pooled barcoded samples, sheared DNA to 400 bp using a Covaris LE220, and enriched for fragments containing the ligated adaptor using a streptavidin bead‐binding assay (Invitrogen Dynabeads M‐280). We prepared libraries for sequencing using the NEBNext Ultra II DNA Library Preparation Kit (New England Biolabs) manufacturer protocol and performed size selection for fragments 300–400 bp in length using Agencourt AMPure XP magnetic beads (Beckman Coulter). We standardized final libraries to 10 nM and performed paired‐end sequencing (2 × 150nt) on an Illumina HiSeq2500 or NovaSeq6000 at the Princeton University Lewis Sigler Genomics Core Facility.

After sequencing, we aligned all forward and reverse reads with the restriction enzyme cut site using a custom perl script (Data S1). We then used *STACKS v1.42* (Catchen et al., 2013) to demultiplex reads, remove reads with >2 bp barcode mismatches or quality scores <90% (using a sliding window of 15% read length), and filter out PCR duplicates using default parameters in *clone_filter*. We manually removed samples with <500,000 reads and performed paired‐end alignment to the reference domestic dog *CanFam3.1* genome (Lindblad‐Toh et al., 2005) with *STAMPY v1.0.21* (Lunter & Goodson, 2011). We used *Samtools v0.1.18* (Li et al., 2009) to sort and filter mapped reads for quality scores (MAPQ ≥ 96) and convert files to BAM format.

We then implemented the *gstacks* and *populations* modules in *STACKS v2.2* (Rochette et al., 2019) to genotype and filter genome‐wide single nucleotide polymorphisms (SNPs) from paired‐end data using the Marukilow model (Maruki & Lynch, 2017). We ran *gstacks* using a dataset of 32 samples representing 24 unique individuals (*N.B*., we excluded wolf 949M from these analyses due to microbial sampling after death). We manually determined which duplicate samples exhibited higher read counts and implemented *populations* using 24 unique samples and the filtering parameters *‐‐write_single_snp* (which only retains one SNP per read) and *–r 0.9* (which only retains loci genotyped in >90% of wolves). This yielded a dataset of 116,953 variant sites genotyped in 24 wolves. We subsequently removed singletons, doubletons, and X‐chromosome sites (due to our mixed‐sex sample set; Clayton, 2009) using *VCFtools v0.1.12b* (Danecek et al., 2011). This produced a dataset of 86,545 high‐confidence autosomal SNPs found throughout the genome.

We additionally created a heavily filtered, pedigree‐informative dataset using *PLINK* (Purcell et al., 2007) to enable pairwise relatedness estimation. This dataset only included biallelic SNPs in Hardy–Weinberg equilibrium (*‐‐hwe 0.001*) with minor allele frequency >0.45 (*‐‐maf 0.45*), following recommended guidelines (Huisman, 2017). It further excluded loci exhibiting statistical linkage disequilibrium as evidenced by genotypic correlation (*‐‐indep‐pairwise 50 5 0*.*2*). After filtering, the pedigree‐informative dataset retained 517 highly informative SNPs genotyped in 24 wolves. As results obtained using the 86,545 SNP and 517 SNP datasets were largely congruent, we primarily present results using the 517 SNP data (see Appendix S1 for 86,545 SNP results).

To consider familial relationships, we calculated pairwise relatedness coefficients in the *R* package *related* (Pew et al., 2015). We used the coancestry function and implemented the dyadic likelihood estimator (*dyadml* *= 1*; Milligan, 2003) with allowance for inbreeding (*allow.inbreeding* *= TRUE*). We additionally identified putative parents and grandparents of wolves with microbiome data by referencing the full YNP wolf pedigree, which included 871 parent–offspring pairs as of October 2020 (vonHoldt et al., 2020).

### 2.3 Microbial DNA extraction and amplicon sequencing

We used a modified Qiagen DNeasy PowerSoil Kit protocol (Qiagen Inc.) to extract DNA from each sample, as described in (DeCandia et al., 2019, 2020). Briefly, we placed swab tips or fecal material into PowerBead tubes that were shaken for two cycles on a Qiagen TissueLyserII. Each cycle lasted for 12 min at 20 shakes/s, with the addition of 60 μl of C1 solution occurring between cycles. We then followed the standard manufacturer protocol until the final elution, when we incubated samples for 10–15 min at room temperature with 60 μl C6 buffer preheated to 70°C. We used sterile swab tips as negative controls during extraction and subsequent library preparation and sequencing. We concentrated extracts to 20 μl in a Vacufuge if needed, quantified DNA using a high‐sensitivity Qubit™ fluorometer, standardized high‐yield samples to 2.5 ng/μl, and included low‐yield samples with concentrations as low as 0.062 ng/μl.

We used barcoded forward (GTGCCAGCMGCCGCGGTAA) and reverse (TAATCTWTGGGVHCATCAGG) primers described in (Caporaso et al., 2011) to amplify and tag the 16S ribosomal RNA (rRNA) V4 region. PCR reactions included 5 μl HiFi HotStart ReadyMix (KAPA Biosystems), 3.2 μl primer mix (1.25 μM), and 1.8 μl template DNA and cycling conditions included: initial denaturation of 94°C/3 min, touchdown cycling for 30 cycles of (94°C/45 s, 80–50°C/60 s, 72°C/90 s) decreasing 1°C each cycle, 12 cycles of (94°C/45 s, 50°C/60 s, 72°C/90 s), and final extension of 72°C/10 min. We quantified PCR product using Quant‐iT™ PicoGreen™ dsDNA assays, pooled equal nanograms of uniquely barcoded libraries, and completed a size selection for fragments between 300 and 400nt using Agencourt AMPure XP magnetic beads. We performed paired‐end sequencing (2 × 150nt) on an Illumina MiSeq in the Princeton University Lewis Sigler Genomics Core Facility.

We sequenced 133 samples (axilla, *n* = 15; dorsal flank, *n* = 20; ear canal, *n* = 21; lip commissure, *n* = 24; nostril, *n* = 21; perianal area, *n* = 25; feces, *n* = 2; negative controls, *n* = 5) collected from 25 wolves (1–11 samples/wolf, with median = 5 and mode = 6). We used a barcode splitter for paired‐end, dual‐indexed data in the online platform Galaxy (Afgan et al., 2018) to demultiplex raw sequencing reads with allowance for a single nucleotide mismatch in sequence tags. We filtered 4,998,642 demultiplexed reads in *QIIME 2 v2020.8* (Bolyen et al., 2019; https://qiime2.org) using the *dada2 denoise‐paired* plugin (Callahan et al., 2016), which corrects probable sequencing errors, trims low‐quality bases, merges paired‐end reads, and removes chimeric sequences. We retained 4,272,465 sequences containing 8,944 amplicon sequence variants (ASVs; Table S1).

We found that negative controls on average contained two orders of magnitude fewer reads (mean *± SE*, 625.60* ± *338.25) than microbiome samples (33,354.20 ± 1,393.65). Negative control reads included 131 ASVs, 127 of which only appeared in one control sample (Table S2). Since frequencies ranged from 2 to 255, we removed ASVs with frequencies lower than 260 from our denoised dataset. This enabled us to mitigate possible contamination while retaining biologically meaningful features (Eisenhofer et al., 2019; Salter et al., 2014). We subsequently removed repeat samples (*n* = 11), samples collected after death (*n* = 6), and negative controls (*n* = 5) for a final dataset of 3,592,905 sequences and 955 ASVs. This included 113 samples (axilla, *n* = 13; dorsal flank, *n* = 17; ear canal, *n* = 19; lip commissure, *n* = 22; nostril, *n* = 18; perianal area, *n* = 22; feces, *n* = 2; Table 1) collected from 24 wolves (1–6 samples/wolf, with median = 5 and mode = 6; Table S4).

**TABLE 1:** Samples included in the microbiome dataset

Sampling pack	Wolves	Axilla	Flank	Ear Canal	Lip	Nostril	Anus	Feces	Total
1108M group	2	0	0	1	2	2	2	1	8
8 Mile	5	4	5	5	5	3	5	0	27
Alone	1	0	0	1	1	1	1	0	4
Cougar Creek	2	0	1	1	1	1	2	0	6
Junction Butte	6	6	6	5	6	4	6	0	33
Wapiti Lake	8	3	5	6	7	7	6	1	35
Total	24	13	17	19	22	18	22	2	113

### 2.4 Characterizing the host‐associated microbiome across multiple body sites

We characterized microbial communities inhabiting each body site using *QIIME 2* (Bolyen et al., 2019; https://qiime2.org). For these analyses, we employed four measures of bacterial diversity to consider different aspects of microbial community composition (Knight et al., 2018). For alpha (or within sample) diversity, we measured microbial species richness using observed amplicon sequence variants (ASVs; Hagerty et al., 2020) and species equitability using Pielou's evenness (Pielou, 1966). For beta (or between sample) diversity, we used qualitative (unweighted UniFrac) and quantitative (Bray–Curtis dissimilarity) measures to consider differences in species presence and abundance (Lozupone et al., 2007). Unweighted UniFrac is a qualitative measure that calculates the amount of branch length in a phylogenetic tree of ASVs leading to unique members of each microbial community (Lozupone & Knight, 2005). This measure primarily considers species presence and ignores the relative abundance of ASVs. In contrast, Bray–Curtis dissimilarity is a quantitative measure that directly incorporates abundance into its calculations (Bray & Curtis, 1957). By concurrently examining observed ASVs, Pielou's evenness, unweighted UniFrac distances, and Bray–Curtis dissimilarity, we were able to compare microbial species richness, evenness, presence, and abundance across body sites.

We calculated these measures using the *core‐metrics‐phylogenetic* and *alpha‐rarefaction* functions in *QIIME 2*. We employed rarefaction to control for different sequencing depths and calculated unweighted UniFrac distances using a midpoint‐rooted phylogeny constructed using the *alignment* and *phylogeny* functions. We used Kruskal–Wallis tests (Kruskal & Wallis, 1952) implemented through the *alpha‐group‐significance* function to compare observed ASVs and Pielou's evenness across body sites. To assess differences in beta diversity, we implemented *PERMANOVA* (Anderson, 2001) through the *diversity adonis* function (Oksanen et al., 2019) and performed principal coordinate analysis (PCoA) using the EMPeror plugin in *QIIME 2* (Vázquez‐Baeza et al., 2013).

In order to assign taxonomy to ASVs, we used reference sequences from the *Greengenes 13_8* database to train a Naive Bayes classifier (Bokulich et al., 2018; DeSantis et al., 2006). Reference sequences were trimmed to match our target amplicon sequence, clustered at 99% similarity, and compared against representative sequences in our dataset using the *classify‐sklearn* function in the *feature‐classifier* plugin for *QIIME 2*. We subsequently clustered body sites hierarchically using Euclidean distances and the average linkage method. We reported the most abundant orders colonizing each body site to characterize high‐level taxonomy and visualize broad‐scale patterns of taxonomic diversity. We additionally considered lower‐level taxonomy by implementing the linear discriminant analysis (LDA) effect size (LEfSe) method in Galaxy with sequences grouped at the genus level (Segata et al., 2011). This method identifies microbial clades (e.g., genera) that underlie differences between two or more biological groups (e.g., body sites). We used default parameter settings and the one‐against‐all strategy for multigroup analysis. Following LEfSe guidelines, we excluded the two fecal samples due to small within‐group sample size (Segata et al., 2011).

### 2.5 Assessing environmental, demographic, and genetic drivers of microbiome composition

We created two subsets of the microbiome data to perform body site‐specific analyses. The first subset included perianal samples (*n* = 22) to represent the gut microbiome (Bassis et al., 2017) and the second subset included dorsal flank samples (*n* = 17) to represent the skin microbiome (Kong et al., 2017). We then considered potential environmental, demographic, and genetic drivers of microbial community composition. We tested for significant associations with age class, body condition, coat color, field season, sampling pack, and sex using single‐ and multifactor *PERMANOVA* implemented through the *diversity adonis* function in *QIIME 2* (Anderson, 2001; Oksanen et al., 2019). We note that the variable field season was confounded with sequencing plate; thus, any observed differences may represent true signal of temporal change or artificial signal of technical batch effect.

We additionally tested for correlations between host genetic distance and microbial dissimilarity using full and partial Mantel tests implemented in the *R* package *vegan* with 999 permutations (Oksanen et al., 2019). Host genetic distances (Euclidean) were calculated using the *dist* function in the *R* package *adegenet* (Jombart, 2008; Jombart & Ahmed, 2011), and microbial distances were calculated in *QIIME 2* using Bray–Curtis dissimilarity and unweighted UniFrac distances. We additionally constructed a dissimilarity matrix for pack membership (where 0 = same pack, 1 = different pack) to implement partial Mantel tests that controlled for shared sampling pack. The test statistic *r* was based on Spearman's rank correlation *rho*, and statistical significance was assessed using a threshold of *p* < .05.

## 3 RESULTS

### 3.1 Genomic analyses support multiple pedigree relationships across sampling packs

**FIGURE 2:**
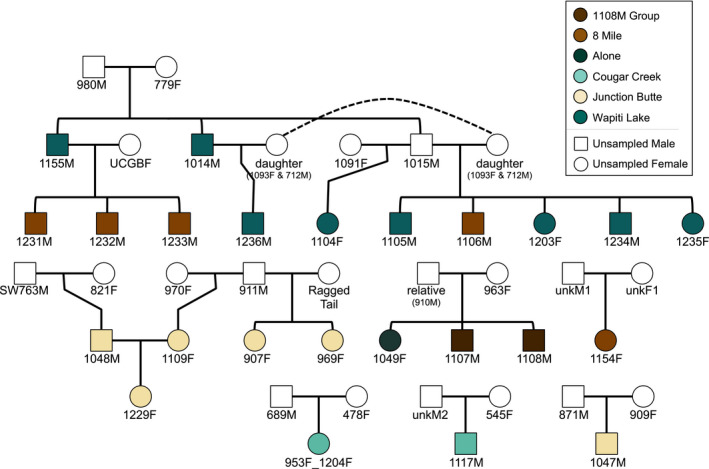
Pedigree relationships for the 24 wolves included in this study (shaded) and their parents. Colors correspond to sampling pack, and dashed lines connect the same wolf in disparate parts of the pedigree

Pairwise relatedness estimates ranged from 0 between putatively unrelated wolves to 0.484 between first‐order relatives (such as parent‐offspring and full sibling pairs). Although relatedness within packs was significantly higher than between packs (*t* test, *t* = 5.95, *p* < .001; Table S3), numerous outliers represent relatives with different pack membership (Figure S2). Contextualizing these 24 wolves within the larger‐scale YNP wolf pedigree (vonHoldt et al., 2020) further supported the presence of parent‐offspring, full sibling, half sibling, avuncular, and other familial relationships in the dataset (Figure 2; Figure S3), leading to a range of genetic relatedness values across shared and unshared environments. These results matched observational data of wolf dispersal. For example, wolves 1108M and 1107M dispersed from their natal pack (8 Mile) 6–8 weeks before being sampled in their newly formed group (1108M Group), whereas wolf 1047M dispersed from his natal pack (8 Mile) four years prior to being sampled in the Junction Butte pack. In further contrast, wolves 1154F, 1231M, 1232M, and 1233M remained in their natal pack (8 Mile) for the duration of this study.

### 3.2 Different body sites exhibit distinct microbial communities

**FIGURE 3:**
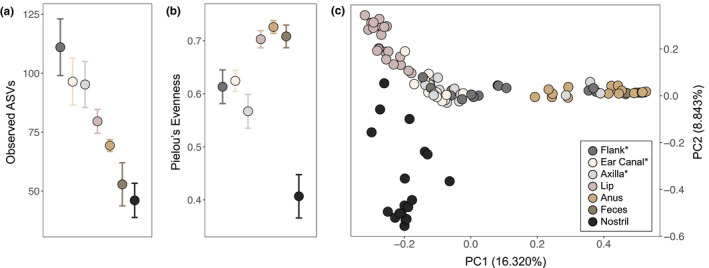
Alpha and beta diversity significantly differed by body site. Mean and standard error for (a) observed ASVs and (b) Pielou's evenness rarefied to 4,600 sequences. (c) The first two PCs calculated using Bray–Curtis dissimilarity. Asterisks indicate heavily and moderately haired body sites

Alpha and beta diversity significantly differed between body sites using multiple diversity metrics. Examination of observed ASVs (Kruskal–Wallis test; *H* = 33.83, *df* = 6, *p* < .001) revealed higher species richness at heavily haired body sites and lower species richness at mucosal and fecal sites (Figure 3a). This pattern was largely driven by significantly lower diversity in mucosal nostril and perianal swabs when compared to haired dorsal flank and axilla swabs (Benjamini–Hochberg corrected *q‐*values < 0.05; Table S5). We also observed significant differences in species equitability between body sites using Pielou's evenness (*H* = 48.144, *df* = 6, *p* < .001), with high equitability observed in fecal and lip communities, moderate equitability at haired sites, and low equitability in the nostril (Figure 3b). Pairwise comparisons returned significant differences between perianal swabs and other body sites (except feces and lip), and nostril swabs and other body sites (except feces; *q‐*values < 0.05; Table S6).

Regarding beta diversity, we observed significant differences in bacterial species abundance (Bray–Curtis dissimilarity; *PERMANOVA*, *R* ^2^ = .281, *df* = 6, *p* = .001) and presence (unweighted UniFrac distances; *R* ^2^ = .403, *df* = 6, *p* = .001) between body sites. Principal coordinate analysis revealed sample clustering by body site, with differences in centroid position and dispersion evident (Figure 3c; Figure S4). These patterns were supported by hierarchical clustering of body sites using Euclidean distances, where perianal and fecal samples formed one group, mucosal lip and nostril samples formed another, and haired axilla, dorsal flank, and ear canal samples formed the third (Figure S5). This evidence suggests that microbial species richness, evenness, presence, and abundance all contribute to the body site‐specific patterns of diversity observed in YNP wolf microbiomes.

**FIGURE 4:**
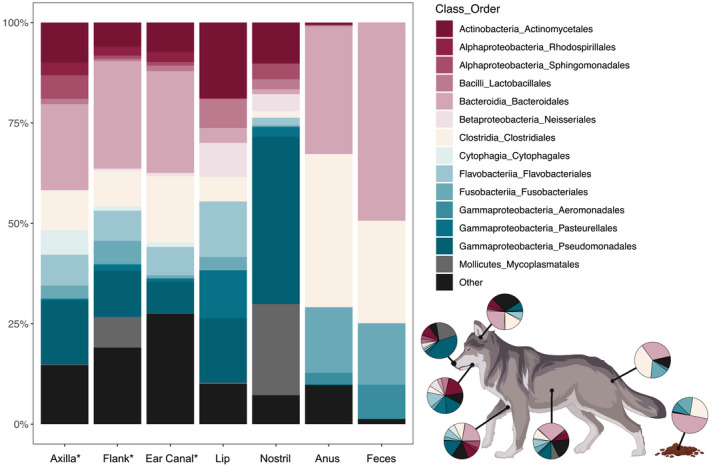
Taxonomic composition of each body site at the order level. Asterisks indicate heavily and moderately haired body sites. Figure created with BioRender

The taxonomic composition of each body site mirrored these patterns (Figure 4). Heavily and moderately haired axilla, dorsal flank, and ear canal samples had high relative abundances of orders Bacteroidales (phylum Bacteroidetes; mean ± *SE* ranged from 18.1 ± 6.3% to 27.6 ± 4.9%), Actinomycetales (phylum Actinobacteria; ranged from 8.3 ± 2.5% to 18.3 ± 7.7%), Clostridiales (phylum Firmicutes; ranged from 9.0 ± 1.9% to 15.3 ± 3.1%), and Pseudomonadales (phylum Proteobacteria; ranged from 6.5 ± 2.2% to 14.1 ± 5.0%). While these taxa were present at mucosal sites and mucocutaneous junctions, microbial communities inhabiting nonhaired sites proved to be more specialized. Lip commissure samples exhibited high relative abundance of Actinomycetales (18.9 ± 3.5%) and Pseudomonadales (16.0 ± 2.3%), but also had high relative abundances of Flavobacteriales (phylum Bacteroidetes; 13.5 ± 1.6%) and Pasteurellales (phylum Proteobacteria; 12.3 ± 2.5%). In contrast to these fairly even relative abundances, nostril, perianal, and fecal samples exhibited taxonomic skews toward one or more microbial taxa. Nostril samples exhibited high relative abundance of Pseudomonadales (44.9 ± 6.0%), whereas perianal (PA) and fecal (F) communities were dominated by Bacteroidales (PA=34.6 ± 4.2%; F = 50.4 ± 15.9), Clostridiales (PA = 35.4 ± 3.5%; F = 24.7 ± 11.7%), and Fusobacteria (phylum Fusobacteria; PA = 16.2 ± 1.5%; F = 14.9 ± 5.2%).

**FIGURE 5:**
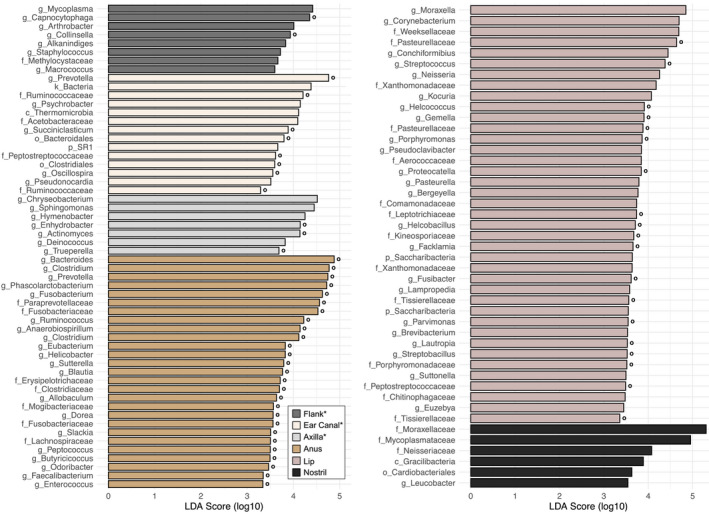
Linear discriminant analysis effect sizes for taxa underlying body site differences, where k = kingdom, p = phylum, c = class, o = order, f = family, and g = genus. Asterisks indicate heavily and moderately haired body sites. Open circles denote predominantly anaerobic clades

Analyses at the genus level (when known) provided finer‐scale insights into the taxonomic composition of each body site. While the overall pattern remained the same (Figure S6), LEfSe revealed which bacterial players most likely drive differentiation between body sites (Figure 5). This analysis returned 7–14 clades abundant in each of the heavily and moderately haired body sites, with 27 clades abundant in perianal samples, 39 abundant in lip commissure samples, and six abundant in nostril samples (all logarithmic LDA scores >3.290, all *p* < .006). We annotated each clade as predominantly aerobic or anaerobic to consider the influence on microenvironment on bacterial communities. This revealed a continuum across body sites, where nostril samples hosted exclusively aerobic clades (with one unknown) and an increasing proportion of anaerobic clades were observed in dorsal flank (25.0%), axilla (42.9%), lip commissure (48.7%), ear canal (57.1%), and perianal (100.0%) samples (*N.B*., the seven clades annotated as “unknown” were combined with aerobic bacteria for these calculations). Dominant clades within the two extremes included aerobic families Moraxellaceae (phylum Proteobacteria), Mycoplasmataceae (phylum Firmicutes), and Neisseriaceae (phylum Proteobacteria) in nostril samples and anaerobic genera *Bacteroides* (phylum Bacteroidetes), *Clostridium* (phylum Firmicutes), *Prevotella* (phylum Bacteroidetes), *Phascolarctobacterium* (phylum Firmicutes), and *Fusobacterium* (phylum Fusobacteria) in perianal samples.

### 3.3 Sampling pack and genetic relatedness are consistently associated with gut and skin microbial communities

Single‐factor *PERMANOVA* of perianal samples (Table S7) consistently returned sampling pack as the variable explaining the most variance within gut microbial communities. This result was statistically significant using both Bray–Curtis dissimilarity (*R* ^2^ = .423, *df* = 5, *p* = .001) and unweighted UniFrac distances (*R* ^2^ = .317, *df* = 5, *p* = .015). Body condition (Bray–Curtis, *R* ^2^ = .304, *df* = 4, *p* = .008) and field season (unweighted UniFrac, *R* ^2^ = .154, *df* = 2, *p* = .018) similarly explained large proportions of variance, but these results were only significant using one diversity measure each. Multifactor *PERMANOVA* containing all six explanatory variables (Table 2; Table S8) supported sampling pack as the only variable significantly predictive of gut microbial community composition using either diversity measure (Bray–Curtis, *R* ^2^ = .423, *df* = 5, *p* = .004; unweighted UniFrac, *R* ^2^ = .317, *df* = 5, *p* = .030; Figure S7).

**TABLE 2:** Results from multifactor *PERMANOVA* implemented with Bray–Curtis (BC) dissimilarity and unweighted UniFrac (UU) distance matrices for gut and skin microbiota

Variable	Gut microbiota			Skin microbiota		
	*df*	BC	UU	*df*	BC	UU
Sampling pack	5	.423*	.317*	3	.208*	.256*
Body condition	4	.133	.172	2	.123	.089
Field season	2	.092	.118	2	.139	.195*
Age class	2	.075	.076	2	.116	.087
Coat color	1	.028	.031	1	.072*	.063
Sex	1	.013	.026	1	.058	.062
Residuals	6	.237	.259	5	.284	.247
Total	21	1.000	1.000	16	1.000	1.000

Note

Degrees of freedom (*df*) and *R* ^2^ values are provided, with asterisks indicating statistical significance (*p* < .05).

Single‐factor *PERMANOVA* of dorsal flank samples (Table S7) similarly returned sampling pack as the variable explaining the most variance within skin microbial communities, although this result was only statistically significant using unweighted UniFrac distances (*R* ^2^ = .256, *df* = 3, *p* = .007). Field season was also significantly associated with species presence (unweighted UniFrac, *R* ^2^ = .202, *df* = 2, *p* = .001) in single‐factor analyses. Multifactor *PERMANOVA* containing all six explanatory variables (Table 2; Table S8) returned sampling pack (Bray–Curtis, *R* ^2^ = .208, *df* = 3, *p* = .039; unweighted UniFrac, *R* ^2^ = .256, *df* = 3, *p* = .002), field season (unweighted UniFrac, *df* = 2, *R* ^2^ = .195, *p* = .002), and coat color (Bray–Curtis, *R* ^2^ = .072, *df* = 1, *p* = .046) as significant predictors underlying skin microbial communities. As with gut microbiota, sampling pack consistently and significantly explained the greatest proportion of variance in skin microbiota using both distance measures (Figure S7).

**FIGURE 6:**
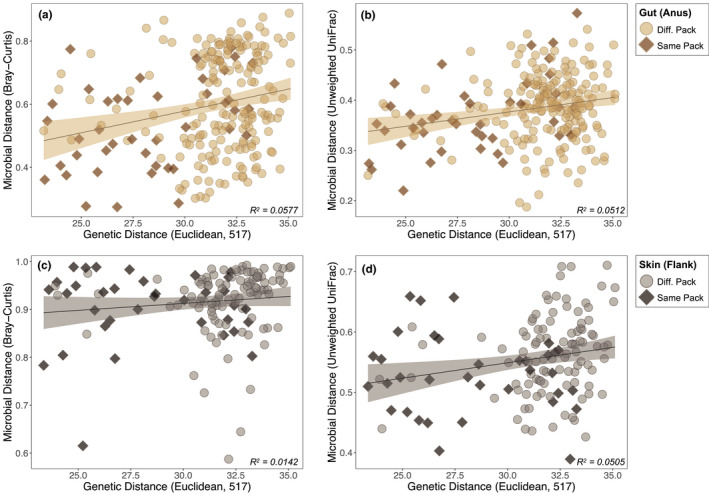
Scatter plots and regression lines of pairwise genetic (Euclidean) and microbial (Bray–Curtis and unweighted UniFrac) distances calculated for (a, b) gut and (c, d) skin microbiota

We additionally observed significant relationships between host genetic and microbial distance. Perianal swabs exhibited a significantly positive correlation between genetic (517 SNP) and microbial community distance (Mantel test; Bray–Curtis, *r* = .25, *p* = .004; unweighted UniFrac, *r* = .17, *p* = .018; Figure 6a,b), even when controlling for sampling pack (partial Mantel test; Bray–Curtis, *r* = .19, *p* = .010; unweighted UniFrac, *r* = .12, *p* = .052; Table S9). In contrast, dorsal flank samples exhibited significant and near‐significant correlations in full Mantel tests (Bray–Curtis, *r* = .20, *p* = .062; unweighted UniFrac, *r* = .25, *p* = .026; Figure 6c,d), but not in partial Mantel tests that controlled for sampling pack (Bray–Curtis, *r* = .17, *p* = .108; unweighted UniFrac, *r* = .17, *p* =.091; Table S9). Similar results were obtained with the 86,545 SNP dataset for perianal samples, and no statistically significant results were obtained for dorsal flank samples (Table S9).

## 4 DISCUSSION

In the present study, we characterized host‐associated bacterial communities inhabiting haired and mucosal body sites of gray wolves sampled in situ at Yellowstone National Park. Consistent with our hypotheses, we reported distinct microbial communities colonizing each body site, with haired sites hosting high alpha diversity and gut samples harboring the largest proportion of anaerobic bacteria. We additionally contextualized skin and gut microbiota within genetic, demographic, and environmental variables to identify the primary factors underlying microbial community presence and abundance. We found that social environment and host genetics were most consistently associated with microbial composition at both body sites. We additionally reported evidence of body condition influencing gut microbiota, and coat color influencing skin microbiota. These results advance the field of microbial natural history and contribute to our understanding of the evolutionary history, ecology, and conservation of gray wolves and their associated microbes.

Examination of resident phyla enabled high‐level contextualization of wolf‐associated microbiomes within Canidae (Table 3). Across wild and captive gray wolves (Wu et al., 2017), red wolves (Bragg et al., 2020), coyotes (*Canis* *latrans*; Colborn et al., 2020; DeCandia et al., 2019; Sugden et al., 2020), red foxes (*Vulpes vulpes*; DeCandia et al., 2019), gray foxes (*Urocyon* *cinereoargenteus*; DeCandia et al., 2019), island foxes (DeCandia et al., 2020), and domestic dogs (Rodrigues Hoffmann et al., 2014), we found the same dominant phyla: Actinobacteria, Bacteroidetes, Firmicutes, Proteobacteria, and Fusobacteria. The first four phyla are considered the core mammalian microbiome and have been documented in humans (Grice & Segre, 2011) and nonhuman mammals (Nishida & Ochman, 2018). Additionally, Fusobacteria has been associated with predatory mammals (Nishida & Ochman, 2018), including marine carnivores and domestic dogs (Nelson et al., 2013). These broad‐scale patterns within Canidae may consequently result from shared evolutionary history or ecology (such as dietary niche; Nishida & Ochman, 2018; Ross et al., 2018).

**TABLE 3:** Dominant phyla (ranked 1–5 from highest to lowest relative abundance) inhabiting Canidae species include Actinobacteria (Act.), Bacteroidetes (Bact.), Firmicutes (Firm.), Fusobacteria (Fuso.), and Proteobacteria (Prot.)

Study	Species	Location	Status^a^	Site^b^	*N*	Act.	Bact.	Firm.	Fuso.	Prot.
Present study	*Canis lupus*	USA	W	A	13	4	1	3	—	2
—	—	—	—	DF	17	4	1	3	—	2
—	—	—	—	EC	19	4	1	2	—	3
—	—	—	—	LC	22	2	3	4	5	1
—	—	—	—	N	18	3	5	4	—	1
—	—	—	—	PA	22	5	2	1	3	4
—	—	—	—	Scat	2	5	1	2	3	4
Wu et al. (2017)	*Canis lupus*	China	C_M_	Scat	14	4	2	1	5	3
Bragg et al. (2020)	*Canis rufus*	USA	W	Scat	2	5	3	1	2	4
—	—	—	C_M_	Scat	3	5	2	3	1	4
—	—	—	C_M,K_	Scat	10	4	3	1	2	5
—	—	—	C_K_	Scat	34	5	2	1	3	4
Rodrigues Hoffmann et al. (2014)	*Canis familiaris*	USA	C_U_	A	12	3	2	4	—	1
—	—	—	—	PA	12	5	3	2	4	1
Colborn et al. (2020)^c^	*Canis latrans*	USA	W	Scat	58	—	1	—	2	—
Sugden et al. (2020)	*Canis latrans*	Canada	W	IN	10	5	4	1	2	3
DeCandia et al. (2019)	*Canis latrans*	USA	R	Skin	4	2	3	4	—	1
—	*Vulpes vulpes*	—	—	Skin	5	3	4	1	5	2
—	*Urocyon cinereoargenteus*	—	—	Skin	1	2	1	—	4	3
DeCandia et al. (2020)	*Urocyon littoralis*	USA	W	A	9	4	2	1	5	3
—	—	—	—	EC	43	2	4	1	—	3
—	—	—	—	EE	31	4	3	1	—	2
—	—	—	—	LC	29	5	2	3	4	1
—	—	—	—	N	15	5	3	1	—	2
—	—	—	—	PA	30	4	1	2	5	3

The different gray shades serve as a simplified heat map—the lower the rank (indicating higher abundance), the darker the shade of gray.

^a^ Status indicates whether samples were collected in the wild (W), at rehabilitation centers (R), or in captivity with meat (C_M_), kibble (C_K_), or unknown (C_U_) diet.

^b^ Body sites include axilla (A), dorsal flank (DF), ear canal (EC), external ear (EE), intestines (IN), lip commissure (LC), nostril (N), perianal area (PA), scat, or skin.

^c^ Taxonomic composition provided at genus level.

In addition to high‐level taxonomy, examination of microbial diversity and genera unique to each body site provides finer‐scale insights into the form and function of commensal microbes. As seen in humans (Grice & Segre, 2011; The Human Microbiome Project Consortium, 2012), gray wolves harbor distinct microbiota at different body sites, consistent with differences in microenvironment and local physiological processes. Increased alpha diversity in haired versus mucosal skin mirrored previous studies conducted in domestic cats (*Felis catus*; Older et al., 2017), domestic dogs (Rodrigues Hoffmann et al., 2014), and Santa Catalina Island foxes (DeCandia et al., 2020), and may result from increased exposure to exogenous environmental factors. In contrast, lower species richness in mucosal sites suggests colonization by microbes specifically adapted to local conditions, as seen in sebaceous human skin (Grice & Segre, 2011). At the genus and family levels, high proportions of aerobic bacteria in wolf nostrils and anaerobic bacteria in wolf guts suggest that oxygen exposure may contribute to differences between respiratory and gastrointestinal microbiota (Dickson & Huffnagle, 2015; Huffnagle et al., 2017; Jalili‐Firoozinezhad et al., 2019; Thursby & Juge, 2017).

These microbes may further function in body site‐specific processes. For example, dominant nostril families Moraxellaceae, Mycoplasmataceae, and Neisseriaceae have been isolated from the noses of domestic dogs and cats, with imbalances associated with nasal disease (Dorn et al., 2017; Tress et al., 2017). Similarly, dominant perianal genera exhibit numerous digestive and health‐promoting properties. For example, *Bacteroides* and *Clostridium* spp. function in bile acid metabolism and digestion of animal proteins and saturated fats (Deng & Swanson, 2015). Although associated with gastrointestinal disease in humans (Hussan et al., 2017), *Fusobacterium* spp. likely contribute to the breakdown of amino acids in healthy dogs and other mammalian carnivores (Pilla & Suchodolski, 2020; Vázquez‐Baeza et al., 2016; Vital et al., 2015). In addition, *Prevotella* spp. function in glucose metabolism and glycogen storage (Tomova et al., 2019), and *Phascolarctobacterium* spp. function in lipid metabolism (Yang et al., 2020). The short‐chain fatty acids produced by these genera (e.g., butyrate) further possess anti‐inflammatory properties that may promote overall gut health (Tomova et al., 2019).

Given significant differences between body sites, we independently identified factors underlying skin and gut microbiota, respectively. Sampling pack emerged as the variable most consistently associated with microbial presence and abundance at both body sites, explaining 31.7%–42.3% of the variance in gut samples and 20.8%–25.6% of the variance in skin samples. This result is consistent with social microbiome theory (Grieneisen et al., 2017; Sarkar et al., 2020) and gray wolf social ecology (Mech & Boitani, 2003; Stahler et al., 2020), as pack members cooperatively share territory, prey, social contacts, and microbes. Similar patterns have been observed in cohabitating humans (Dill‐McFarland et al., 2019; Song et al., 2013) and nonhuman mammals (Goodfellow et al., 2019; Leclaire et al., 2014; Wikberg et al., 2020) with varying degrees of relatedness.

To disentangle the role of relatedness in these patterns, we leveraged biobanked samples and the highly resolved YNP wolf pedigree (vonHoldt et al., 2020) to evaluate microbial similarity between first‐order relatives, distant relatives, and unrelated dispersers with short (e.g., 1108M and 1107M) and long (e.g., 1047M) histories of dispersal from their natal packs. We found significantly positive correlations between host genetic and microbial distances at both body sites. However, after controlling for shared sampling pack, this result only remained significant for gut samples. This suggests that host genetics influence gut microbes more than skin microbes, perhaps due to differences in environmental exposure or maintenance of specialized microbiota (Grice & Segre, 2011; Thursby & Juge, 2017). Similar patterns have been observed in humans, where cohabitation exerted a stronger effect on the skin microbiome compared to fecal microbiota (Song et al., 2013), as well as in great spotted cuckoos (*Clamator glandarius*) and magpies (*Pica pica*), where shared diet and environment were predictive of esophageal but not cloacal microbiota (Lee et al., 2020).

We observed further differences in the secondary factors underlying gut and skin microbial communities. For example, body condition significantly explained 30.4% of variance in gut microbial species abundance (N.B., this value reduced to 13.3% when controlling for sampling pack). This follows previous studies linking body condition, health, and disease to microbial composition in a variety of contexts, including clinical medicine (Gupta et al., 2020), veterinary medicine (Bradley et al., 2016; Rodrigues Hoffmann et al., 2014), and wildlife rehabilitation and conservation (DeCandia et al., 2019, 2020). We additionally found that coat color significantly explained 7.20% of variance in skin microbial species abundance when controlling for sampling pack. This presented a compelling preliminary result, as the mutation underlying melanism in gray wolves encodes for a beta defensin protein known to regulate microbiota and immune processes at the skin barrier (Anderson et al., 2009; Candille et al., 2007; Meade & O'Farrelly, 2019). Consequently, this mutation may lead to characteristic differences in the skin microbiota of black and gray wolves: an exciting frontier for further study.

Characterizing body site‐specific microbiota and the factors underlying them is an important objective within evolution and ecology. While sampling wildlife populations in situ present logistical challenges, these analyses are critical for informing our understanding of the evolutionary history, ecology, and conservation of hosts and their associated microbes. Regarding evolution and ecology, we found that the core microbiota of YNP wolves mirrors numerous species within Canidae, likely due to shared phylogenetic history and characteristics such as carnivory. We additionally found that different body sites host distinct microbiota, with functions and products directly related to physiological processes (e.g., aiding digestion) and host health (e.g., regulating inflammation). We further found that social environment and host genetics influence skin and gut microbiota, with evidence supporting body condition and coat color as secondary factors influencing body site‐specific microbiota.

Considered together, these results provide important baseline information for the long‐term conservation of gray wolves and related species (Trevelline et al., 2019; West et al., 2019). We can now monitor in situ populations for the emergence of novel pathogens or microbial imbalances that may contribute to morbidity and mortality on the landscape (DeCandia et al., 2018). We can further adjust the diet (e.g., increase meat consumption) and social environment (e.g., enable group living) of captive‐managed gray wolves to promote more natural microbiota. This study represents an important step toward these conservation goals and calls for similar studies to be conducted in additional host–microbe systems in captivity and the wild. Host characteristics such as evolutionary history, genetics, ecology, demography, and environment shape microbiota in innumerable, nuanced ways—and microbiota, in turn, shape their hosts. By characterizing the microbiota inhabiting diverse wildlife systems across body sites and environmental contexts, we can elucidate common patterns and processes that deepen our understanding of these relationships and contribute to successful wildlife conservation efforts.

## CONFLICT OF INTEREST

None declared.

## AUTHOR CONTRIBUTIONS

**Alexandra L. DeCandia:** Conceptualization (equal); Data curation (equal); Formal analysis (lead); Funding acquisition (equal); Methodology (equal); Visualization (lead); Writing‐original draft (lead); Writing‐review & editing (lead). **Kira A. Cassidy:** Data curation (equal); Writing‐review & editing (equal). **Daniel R. Stahler:** Conceptualization (equal); Data curation (equal); Funding acquisition (equal); Methodology (equal); Supervision (equal); Writing‐review & editing (equal). **Erin A. Stahler:** Data curation (equal); Writing‐review & editing (equal). **Bridgett M. vonHoldt:** Conceptualization (equal); Funding acquisition (equal); Methodology (equal); Supervision (equal); Writing‐review & editing (equal).

## Supporting information

Appendix S1

Data S1

## Data Availability

Sequence data are available through NCBI's public Sequence Read Archive (https://www.ncbi.nlm.nih.gov/sra) under BioProject PRJNA732683. Microbiome data are available as demultiplexed forward and reverse FASTQ files (BioSamples SAMN19335927 to SAMN19336039), and RAD sequencing data are available as BAM files mapped to the CanFam3.1 domestic dog genome assembly (BioSamples SAMN19336040 to SAMN19336063).
