# Supplementary material for: Social environment and genetics underlie body site‐specific microbiomes of Yellowstone National Park gray wolves (Canis lupus)
Source: Ecol Evol. 2021 Jun 21;11(14):9472–88. doi: 10.1002/ece3.7767 (PMC8293786; doi:10.1002/ece3.7767)
Supplement: Supplementary file 1 — Appendix S1 [file ECE3-11-9472-s001.docx]

**Social environment and genetics underlie body site specific microbiomes of Yellowstone National Park gray wolves (*Canis lupus*)**

**APPENDIX 1. Supporting Tables and Figures**

**
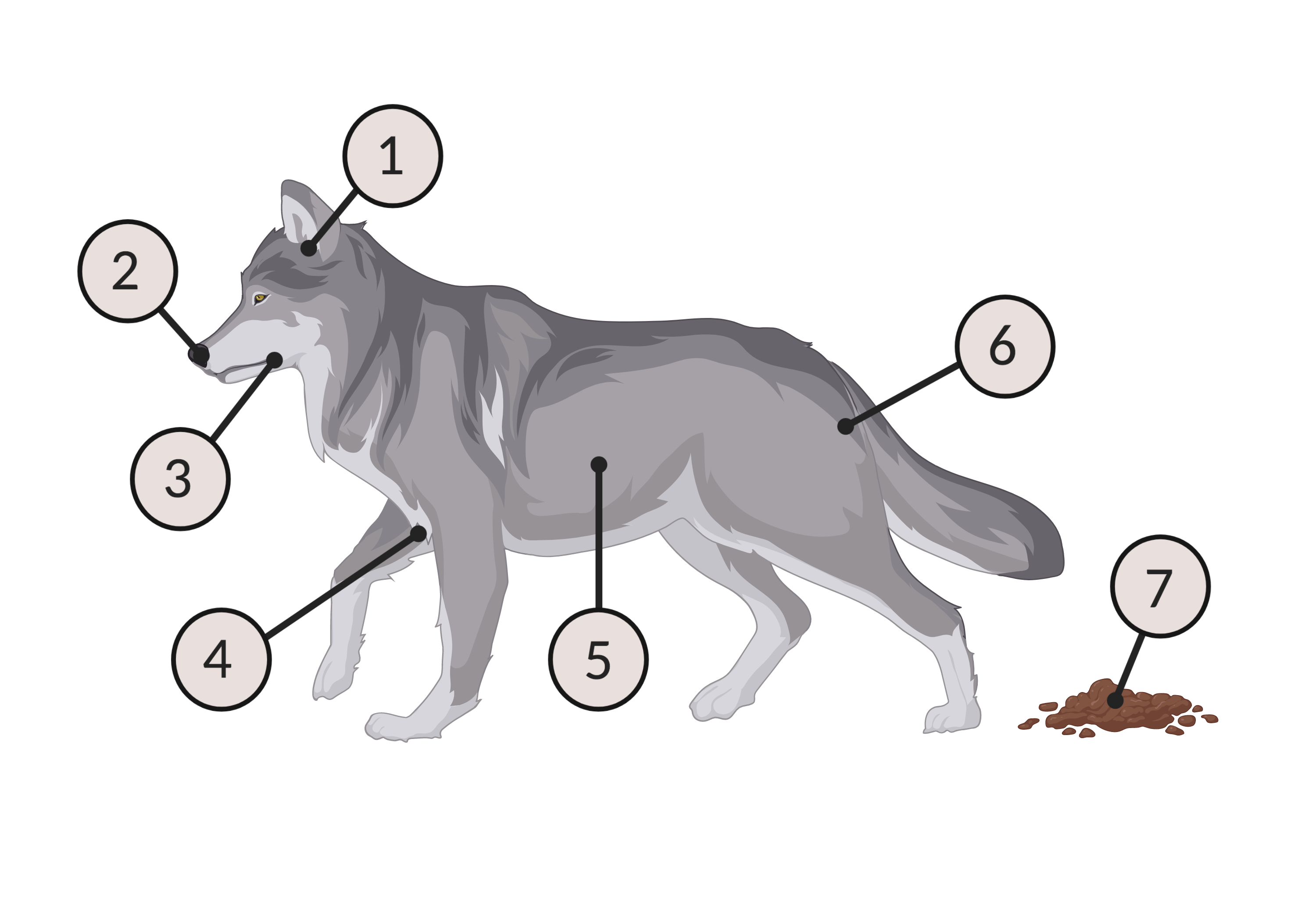
**

**Figure S1.** Body sites swabbed included (1) ear canal, (2) nostril, (3) lip commissure, (4) axilla, (5) dorsal flank, and (6) perianal area. We additionally collected fresh (7) scat samples, as available. Figure created with BioRender.

**Table S1.** Microbial sequences retained after each step of data processing, from the total number of raw sequences generated, followed by quality filtering, denoising, merging of paired-end reads, and removal of chimeras.

| **Sample ID** | **Raw** | **Filtered** | **Denoised** | **Merged** | **Non-chimeric** |
| --- | --- | --- | --- | --- | --- |
| CL9075 | 24787 | 23372 | 22368 | 18398 | 18291 |
| CL9076 | 41282 | 40292 | 40069 | 38906 | 38838 |
| CL9077 | 46552 | 45303 | 45061 | 43672 | 43316 |
| CL9078 | 23372 | 21319 | 20738 | 17920 | 17890 |
| CL9079 | 25472 | 24261 | 23584 | 20872 | 20704 |
| CL9080 | 28653 | 27538 | 27310 | 26148 | 25850 |
| CL9860 | 55758 | 41615 | 41053 | 36994 | 36856 |
| CL9861 | 58443 | 47820 | 47444 | 44127 | 44087 |
| CL9862 | 48871 | 45896 | 45762 | 45179 | 45077 |
| CL9863 | 52443 | 38606 | 37912 | 32192 | 32160 |
| CL9864 | 44793 | 35992 | 35473 | 32173 | 31664 |
| CL9865 | 49357 | 41593 | 41313 | 38868 | 38570 |
| CL9868 | 32451 | 26943 | 26728 | 25191 | 25177 |
| CL9869 | 31918 | 26616 | 26208 | 24936 | 24867 |
| CL9870 | 41992 | 29817 | 29506 | 26552 | 26548 |
| CL9871 | 60482 | 46944 | 46682 | 43460 | 42958 |
| CL9874 | 48362 | 44972 | 44716 | 43345 | 43319 |
| CL9877 | 44663 | 37814 | 37520 | 35630 | 35405 |
| CL9879 | 45354 | 31866 | 31610 | 29392 | 29302 |
| CL9880 | 38429 | 36128 | 36014 | 33177 | 33064 |
| CL9883 | 55576 | 45023 | 44720 | 43102 | 42309 |
| CL9884 | 47762 | 25181 | 24420 | 19864 | 19837 |
| CL9885 | 58682 | 51941 | 51449 | 49369 | 48697 |
| CL9886 | 10935 | 10710 | 10594 | 10178 | 10133 |
| CL9887 | 45780 | 39183 | 38868 | 36962 | 36827 |
| CL9888 | 31975 | 27188 | 26850 | 25423 | 25411 |
| CL9889 | 64661 | 54795 | 54473 | 51714 | 51122 |
| CL9890 | 38631 | 21449 | 20850 | 16643 | 16641 |
| CL9891 | 37635 | 32682 | 32494 | 31425 | 31197 |
| CL9892 | 66765 | 55619 | 55394 | 53646 | 53556 |
| CL9895 | 58354 | 45084 | 44798 | 42666 | 42258 |
| CL9897 | 45202 | 37001 | 36824 | 34711 | 34675 |
| CL9899 | 38628 | 32359 | 31970 | 30373 | 30332 |
| CL9900 | 46886 | 40520 | 40233 | 39104 | 39104 |
| CL9901 | 47679 | 38540 | 38248 | 36762 | 36405 |
| CL9902 | 52219 | 24247 | 23561 | 18401 | 18395 |
| CL9903 | 60926 | 55046 | 54700 | 53095 | 52323 |
| CL9905 | 10062 | 9855 | 9662 | 9306 | 9254 |
| CL9906 | 34987 | 28798 | 28363 | 26647 | 26596 |
| CL9907 | 48258 | 38707 | 38493 | 36602 | 36201 |
| CL9908 | 47730 | 40893 | 40665 | 39229 | 38796 |
| CL9909 | 42860 | 36624 | 36441 | 34632 | 34386 |
| CL11127 | 8063 | 7692 | 7364 | 6420 | 6420 |
| CL11128 | 26116 | 25110 | 24948 | 23762 | 23742 |
| CL11129 | 5776 | 5250 | 5200 | 5077 | 5077 |
| CL11132 | 28712 | 27813 | 27634 | 26531 | 26382 |
| CL11133 | 29095 | 28182 | 27973 | 26718 | 26604 |
| CL11134 | 51104 | 47398 | 46381 | 40145 | 40029 |
| CL11135 | 21106 | 20505 | 20347 | 19724 | 19681 |
| CL11136 | 7751 | 7560 | 7455 | 7250 | 7250 |
| CL11137 | 9371 | 8968 | 8715 | 8035 | 8035 |
| CL11138 | 33022 | 32033 | 31749 | 30354 | 30236 |
| CL11140 | 6787 | 6612 | 6453 | 6094 | 6094 |
| CL11141 | 6064 | 5949 | 5760 | 5347 | 5347 |
| CL11142 | 27247 | 26124 | 25853 | 24562 | 24527 |
| CL11143 | 15378 | 14905 | 14417 | 13380 | 13250 |
| CL11144 | 23040 | 22411 | 22205 | 21496 | 21491 |
| CL11147 | 21368 | 18988 | 18532 | 15364 | 15285 |
| CL11148 | 26460 | 25592 | 25381 | 24493 | 24358 |
| CL11149 | 19925 | 17321 | 16917 | 14880 | 14880 |
| CL11150 | 22791 | 22238 | 21925 | 21143 | 21115 |
| CL11151 | 32646 | 31173 | 31035 | 30022 | 29985 |
| CL11153 | 8769 | 8582 | 8223 | 7391 | 7391 |
| CL11154 | 22705 | 22057 | 21815 | 20865 | 20844 |
| CL11155 | 27357 | 26661 | 26499 | 25757 | 25655 |
| CL11156 | 12928 | 12651 | 12338 | 11857 | 11838 |
| CL11157 | 36951 | 35492 | 35234 | 32866 | 32707 |
| CL11158 | 9528 | 9306 | 9240 | 9109 | 9109 |
| CL11160 | 9245 | 9029 | 8830 | 8529 | 8529 |
| CL11161 | 22233 | 21600 | 21328 | 20262 | 20222 |
| CL12457 | 37851 | 36882 | 36260 | 34600 | 34517 |
| CL12458 | 48102 | 46214 | 45931 | 44277 | 44001 |
| CL12459 | 30407 | 29879 | 29704 | 29109 | 29109 |
| CL12460 | 41940 | 40990 | 40761 | 39610 | 39465 |
| CL12461 | 53651 | 51990 | 51512 | 49496 | 49273 |
| CL12462 | 57335 | 55781 | 55475 | 53982 | 51566 |
| CL12463 | 20499 | 19599 | 19162 | 17946 | 17935 |
| CL12464 | 41698 | 40528 | 40248 | 39248 | 38525 |
| CL12465 | 34105 | 33145 | 33017 | 32713 | 32713 |
| CL12466 | 23643 | 22876 | 22619 | 22084 | 22081 |
| CL12467 | 60913 | 58543 | 58157 | 56593 | 56535 |
| CL12468 | 39363 | 38184 | 37720 | 36266 | 36204 |
| CL12469 | 46123 | 45131 | 44435 | 42096 | 42031 |
| CL12470 | 49189 | 48156 | 47799 | 45683 | 45557 |
| CL12471 | 37461 | 36551 | 36273 | 35176 | 35081 |
| CL12473 | 38912 | 37891 | 37320 | 35302 | 35152 |
| CL12474 | 77421 | 75642 | 75194 | 73306 | 72627 |
| CL12475 | 44915 | 44155 | 43593 | 40623 | 40469 |
| CL12476 | 24699 | 24169 | 24023 | 23253 | 22104 |
| CL12477 | 49155 | 47990 | 47696 | 46169 | 45624 |
| CL12478 | 60622 | 59402 | 58852 | 57068 | 56232 |
| CL12479 | 69663 | 68405 | 67828 | 66306 | 65905 |
| CL12480 | 27691 | 27021 | 26777 | 25967 | 25861 |
| CL12481 | 38648 | 37405 | 36799 | 34893 | 34889 |
| CL12482 | 49286 | 48074 | 47741 | 46338 | 45927 |
| CL12483 | 47819 | 46850 | 46720 | 45803 | 45558 |
| CL12484 | 18300 | 17799 | 17546 | 16319 | 16318 |
| CL12485 | 19165 | 18676 | 18445 | 17944 | 17920 |
| CL12486 | 31340 | 30660 | 30369 | 29564 | 29313 |
| CL12487 | 74172 | 70285 | 69156 | 63364 | 63109 |
| CL12488 | 34099 | 33350 | 33077 | 32224 | 31120 |
| CL12489 | 46429 | 45481 | 45367 | 40704 | 40524 |
| CL12491 | 44050 | 42929 | 42236 | 39276 | 39157 |
| CL12492 | 16330 | 15880 | 15738 | 15354 | 15354 |
| CL12493 | 43598 | 42524 | 41879 | 39676 | 39552 |
| CL12494 | 31463 | 30668 | 30404 | 29546 | 29441 |
| CL12495 | 33567 | 32941 | 32776 | 29856 | 29761 |
| CL12496 | 42485 | 41616 | 41211 | 39574 | 39522 |
| CL12497 | 46996 | 46019 | 45513 | 43966 | 43796 |
| CL12498 | 35085 | 34295 | 33925 | 33045 | 32955 |
| CL12499 | 58087 | 56879 | 56203 | 53429 | 53356 |
| CL12500 | 52825 | 51060 | 50674 | 49175 | 48901 |
| CL12501 | 33763 | 33111 | 32926 | 32106 | 31254 |
| CL12502 | 40095 | 38697 | 38494 | 37571 | 37571 |
| CL12503 | 41200 | 40310 | 40026 | 39047 | 39033 |
| CL12504 | 28591 | 27618 | 27440 | 26753 | 26680 |
| CL12505 | 58091 | 55245 | 54830 | 52283 | 52220 |
| CL12506 | 78352 | 76450 | 75824 | 71915 | 71310 |
| CL12507 | 50456 | 48883 | 48611 | 47858 | 47381 |
| CL12508 | 63517 | 60965 | 60420 | 56881 | 56660 |
| CL12509 | 75510 | 73268 | 72695 | 70577 | 70072 |
| CL12510 | 43983 | 42166 | 41800 | 39977 | 39781 |
| CL12511 | 40493 | 39087 | 38162 | 33923 | 33708 |
| CL12512 | 30957 | 29837 | 29594 | 28599 | 28512 |
| CL12513 | 14341 | 13554 | 13476 | 13373 | 13373 |
| CL12514 | 39543 | 38563 | 38265 | 36556 | 36042 |
| CL12515 | 68359 | 66326 | 65781 | 61355 | 60815 |
| CL12516 | 100545 | 98716 | 98209 | 96069 | 95404 |
| Blank01 | 518 | 465 | 409 | 350 | 350 |
| Blank02 | 977 | 908 | 786 | 625 | 625 |
| Blank03 | 339 | 263 | 192 | 127 | 127 |
| Blank04 | 374 | 251 | 184 | 101 | 101 |
| Blank05 | 2371 | 2259 | 2174 | 1925 | 1925 |
| **Total** | 4998642 | 4572811 | 4527325 | 4299313 | 4272465 |
| **Average** | 37583.774 | 34382.038 | 34040.038 | 32325.662 | 32123.797 |

**Table S2.** Amplicon sequence variants (ASVs) obtained in the five negative control samples included in this study, with total frequency and number of samples indicated.

| **ASV Identifier** | **Frequency** | **No. Samples** |
| --- | --- | --- |
| 3f0449c545626dd14b585e9c7b2d16f4 | 255 | 4 |
| 627114b943a837a895c43e1f430c8d3d | 207 | 1 |
| ec01ad235309b2c3d88b4919069243c5 | 167 | 1 |
| 3f0a5cd9b3240dfaca7af3e86ae24ad2 | 131 | 1 |
| f309ea1ec8287026c5e76ba862929f85 | 130 | 2 |
| a55c76a00f38acb391d3ec56b134a7c4 | 119 | 1 |
| ae3ae4cd1ba0f60f3298fdb16c0acba4 | 104 | 1 |
| 5e9fc9cf92678806672439fe17aa181c | 92 | 1 |
| 17f016e3298748a0eb03b67eb9267a19 | 90 | 3 |
| 83b0bde4963afa2035706cc3fac899e7 | 68 | 1 |
| b4eaabba214836a0720f212d4d3966b9 | 58 | 1 |
| c2d41dc0a7b8eaedcf4697512aee4427 | 53 | 1 |
| 3b331720ec8c05a64bcbc0975acf511a | 52 | 1 |
| dd93bc698bdb8d4a9caa83e404af4bb5 | 51 | 1 |
| eaba6b0dcca801e84a0a5901ae92f462 | 49 | 1 |
| 816b13f71e3eab6f4e0e244410a9f432 | 48 | 1 |
| 8adb5559e135233565acfea1287280bf | 46 | 1 |
| 80bcdf05bcec9712f3a3dff9ffda14e8 | 45 | 1 |
| f2b99a001673871809b58e7c9c61856a | 37 | 3 |
| 17c2e6af6b670e89c5c769cc869f160e | 36 | 1 |
| be3e3f4abf85e1255f46f70853b75a83 | 32 | 1 |
| 966660ca864ac97b9c3fdf05410dec92 | 30 | 1 |
| 96ccada6b82d76566c0674d882866745 | 29 | 1 |
| c0bbb7b792915ca4b048b828440425bb | 26 | 1 |
| cd0fdb0ac5bee0eb1b717b5effd41ef4 | 25 | 1 |
| e16be6de6436803e3b84a5b5a648bb17 | 23 | 1 |
| a997df89c93a418bbc2ffce09318cd57 | 23 | 1 |
| c05b67b25d11955d4dc0c8e06adb870c | 21 | 1 |
| b99ece232f0f43c39c6d18e12136e166 | 20 | 1 |
| de26b2794c12660cc8994a66d08f532b | 20 | 1 |
| 4ec79e602df50e74bcbb1e9d7cb58341 | 20 | 1 |
| 1727823d00b87c3b18e342c912b4c908 | 19 | 1 |
| 724420e29ada99f5ccb2ca259411054d | 19 | 1 |
| 53cddc49fabbdf696366a78df2558656 | 19 | 1 |
| f19f4001aed2b27296977db4c0313e25 | 18 | 1 |
| 8d35b756634b4588349a78a0866b8b94 | 18 | 1 |
| c9a37732d9bcedc950ebcb20ef3c9ab6 | 18 | 1 |
| b89e31020ef3a047590f17dda1a699d9 | 17 | 1 |
| 1aa54d61f344039a9099f86f91fbdca8 | 17 | 1 |
| e9736f477fb55fed52feb9125916c1a8 | 17 | 1 |
| 4865f88c0b7badc418f6421c8327a4a8 | 17 | 1 |
| 1006d42c9984f242af419f88f8f30b61 | 17 | 1 |
| e889c87463a97c5e5db54503335d9b41 | 17 | 1 |
| a723a8e7d20fde47a69c1adae5d23cbb | 16 | 1 |
| 4a8cbd4289458340c478f5d323fe155c | 16 | 1 |
| 8a224c30e7861020ed61faca13561be0 | 16 | 1 |
| 81fb9360f5e5db4f7d99b5f099c691bf | 16 | 1 |
| 5fd6f93ac640d34b0074a52b60e430aa | 15 | 1 |
| 5c78314ff92e6fec9aa07acc1fa0dc24 | 15 | 1 |
| 6744d162b22ad3ab431f9e9efdda81fc | 15 | 1 |
| 1834b94bb96d69405df9aa497dceab4c | 15 | 1 |
| cb6a59fbf6a5e1fed6bfe86ad5d82351 | 15 | 1 |
| e3e89166daa575e51d7a14bc65f11153 | 15 | 1 |
| b0bc1e2c61b1baed3229bf8437f3a6aa | 15 | 1 |
| 61c2ae6c1ea8ff91b08ae219ca4480d1 | 14 | 1 |
| 338307209854f91b45fb13daba661d1d | 14 | 1 |
| 2c554d0440e2ea1cc7beed4bac52cd64 | 14 | 1 |
| 1f520fa03af01586362ae696e5477213 | 14 | 1 |
| d4b63af2bdda482eebb531758316c594 | 13 | 1 |
| 66670f3667ab754fd9a1fb24d0c81ed2 | 13 | 1 |
| e4fa79443ca6f2b8fb79c56bfec6b12a | 13 | 1 |
| ff50ccbd5249cb47f365fc2030f537c7 | 13 | 1 |
| d317ddf4723a65616c36048e746537a4 | 13 | 1 |
| 559c09f6e7424b80a861727b9bd7b7bd | 13 | 1 |
| 1dfc4eff4f012d7e55dd3eedb91cd118 | 12 | 1 |
| 5a90106a71eb6b7dd8fae5fb1b8967c8 | 12 | 1 |
| 375f09f33ba3812f3021f7560cda27c8 | 12 | 1 |
| 15aa70fc481647f82791cb3241c8b21e | 12 | 1 |
| 0d4c0106d317990d851c5683fac4102d | 12 | 1 |
| 5157b6c733f7ada049e04b591344eb65 | 12 | 1 |
| 030d0dfa992345e388f3e3f2b833a3af | 12 | 1 |
| ae92027db22dbe2b0acea9777c22eda3 | 12 | 1 |
| b42308394497a4fc884f5be0630bdd32 | 11 | 1 |
| 52e3cb0935a827c84c1e61f13bbf45d5 | 11 | 1 |
| f5e8254ee50f241b546ecb7ede4b8b3d | 11 | 1 |
| e3d7402dfa7a45343b415c679366e347 | 11 | 1 |
| 668684b118e9c432c4800f21b3181674 | 11 | 1 |
| 1840e3208c0ba374c90750d47438bf38 | 11 | 1 |
| e2e327783ae5df94539aefd9be50b5e3 | 11 | 1 |
| 620edd2956ede81b09704c3f3f5657fa | 11 | 1 |
| 435c5dbbafdabfe7e20490d579a5f6d8 | 11 | 1 |
| 035d1c2d35563476025ea4f288d8065d | 10 | 1 |
| e1bec1c5e4949c0eb7f3ef2ea7b10463 | 10 | 1 |
| 7994ffd9e53c10525b5ae96dbbf71bf4 | 10 | 1 |
| 0c0d7be5fb446c35af4c8908f08e8a42 | 9 | 1 |
| 0dc5e12ca236f832cb19c0216efec505 | 9 | 1 |
| 78f3b5eeb318a06cdb31bbb5ea98aa5c | 9 | 1 |
| 51410f5dc0b31b7abf27cf4ee6f0b7d2 | 9 | 1 |
| 2998d1c6863e6ba15c293025993c786f | 8 | 1 |
| fad7959733a200344fe5a2a9f6252f81 | 8 | 1 |
| 2c6c17a27b1ba5228f6fcc36f5806882 | 8 | 1 |
| 1dfad3e87026bec25ad21b02370ba6ff | 7 | 1 |
| 7b5a2cc644eea5dcd84749a72e2e87b5 | 7 | 1 |
| 343af697bad1edfaea514e1bd5ea64a3 | 7 | 1 |
| 18de6f43344161426986ed3505682c9d | 7 | 1 |
| 3fefffacc895abb4eea3ede9cc442ed3 | 7 | 1 |
| 80ac4217f901083ebed3212899ddba21 | 7 | 1 |
| a4d160cdd7300749c94999485078b5da | 7 | 1 |
| 25acad3dd9a5fed15ac4ffc7a1472e02 | 7 | 1 |
| a42666b23565ad4493adbf6ba96eb347 | 7 | 1 |
| 63361d7acb32510b5322a154f59aca21 | 7 | 1 |
| 9e83221f9cb977a6d0797f4a8e378263 | 7 | 1 |
| eb9de9a9fbca5faf98e2f3abf1f72ac6 | 7 | 1 |
| 7a88ef8a99cd1665ef2bd6878d17023d | 7 | 1 |
| 891347841ebc791583cd917efad53ca9 | 7 | 1 |
| f8119db23244cad00bfddec7a80fb161 | 7 | 1 |
| 814893d3eaa36358bd83df44b211d5cb | 6 | 1 |
| bace8f4427118794414486b15b14692c | 6 | 1 |
| 482de3766dcf1ee281b235bfd988f374 | 6 | 1 |
| 827ded5087c17f9faf76cb4f688a8365 | 6 | 1 |
| 431aeb0e41d60b227956c41ebcb60c7d | 6 | 1 |
| 7f5acda825f580685958e3ccddc5d3cd | 5 | 1 |
| 12255863fbcec24de67c1dd57843814f | 5 | 1 |
| b872c2693cbad1fa99e19284ea1452fe | 5 | 1 |
| d01f7b167db6a87f8a72e3b700f868de | 5 | 1 |
| 24a27b99438f1e0b79ee41e59f7ece55 | 5 | 1 |
| f54b17c150d7c8bc181cbecafe8e1dda | 5 | 1 |
| b6040847db5745437a45be6403a172d0 | 5 | 1 |
| 5fe350f591d1f82ee5f2a0c8a8b9bca8 | 5 | 1 |
| a05bc04c0071563dea902df8248080a9 | 5 | 1 |
| 9927da2609504b3e85ef6e52e3b4c797 | 5 | 1 |
| f0c5ba979c780080f3119fcc12977c4a | 5 | 1 |
| 6e1ee4ac952c619b04c1871f5497b94f | 4 | 1 |
| c93208260b55c64b3e6fce04cc24e2d6 | 4 | 1 |
| 21885106f55cce0e4e6565fdebc32fa2 | 4 | 1 |
| 8774c8f9dea283260be0b811c8ac81a5 | 4 | 1 |
| 8f72b04ed28af5c7340f78a96d3387e4 | 4 | 1 |
| 9ae8263aa315e8bb29e278fa7c5882cf | 3 | 1 |
| 5ea9f66a71d51c69aa08723cb2704e6a | 3 | 1 |
| ea355aea179faee5e1ed5954211f393f | 2 | 1 |
| 1a0b1855c9f3ea22a57f898b23e3de48 | 2 | 1 |

**Table S3.** Summary statistics for pairwise relatedness estimates within and between packs for the full dataset (All) and each individual pack observed: 1108M Group (1108M), 8 Mile (8M), Alone (Alone), Cougar Creek (CC), Junction Butte (JB), and Wapiti Lake (WL). Information provided includes sample size (n), mean value, standard deviation (SD), minimum, maximum, test statistic (*t*), degrees of freedom (*df*), and significance value (*p*).

|  | ***Within Pack*** | | | | ***With Other Packs*** | | | | **t-test results** | | |
| --- | --- | --- | --- | --- | --- | --- | --- | --- | --- | --- | --- |
| **Pack (n)** | **Mean** | **SD** | **Min** | **Max** | **Mean** | **SD** | **Min** | **Max** | ***t*** | ***df*** | ***p*** |
| 1108M (2) | 0.448 | NA | NA | NA | 0.051 | 0.108 | 0.000 | 0.453 | NA | NA | NA |
| 8M (5) | 0.116 | 0.147 | 0.000 | 0.373 | 0.043 | 0.102 | 0.000 | 0.397 | 1.542 | 9.927 | 0.154 |
| Alone (1) | NA | NA | NA | NA | 0.069 | 0.134 | 0.000 | 0.453 | NA | NA | NA |
| CC (2) | 0.159 | NA | NA | NA | 0.027 | 0.070 | 0.000 | 0.279 | NA | NA | NA |
| JB (6) | 0.148 | 0.182 | 0.000 | 0.484 | 0.009 | 0.036 | 0.000 | 0.221 | 2.939 | 14.151 | 0.011 |
| WL (8) | 0.167 | 0.135 | 0.004 | 0.471 | 0.025 | 0.085 | 0.000 | 0.397 | 5.316 | 31.875 | <0.001 |
| *All (24)* | *0.157* | *0.153* | *0.000* | *0.484* | *0.030* | *0.086* | *0.000* | *0.453* | *5.953* | *62.792* | *<0.001* |

**Figure S2.** Boxplots of pairwise relatedness estimates within (blue) and between (purple) packs revealed numerous relatives with different pack membership. Groups depicted in the figure include: all samples (All), 8 Mile (8M), Junction Butte (JB), Wapiti Lake (WL), 1108M Group (1108M), Cougar Creek (CC), and Alone (Alone).

**Figure S3.** Pedigree relationships for the 24 wolves included in this study (shaded) and their parents and grandparents. Colors correspond to sampling pack, and dashed lines connect the same wolf in disparate parts of the pedigree.

**Table S4.** Samples included in the microbiome dataset.

| **Wolf ID** | **Sampling Pack** | **Axilla** | **Flank** | **Ear Canal** | **Lip** | **Nostril** | **Anus** | **Feces** | ***TOTAL*** |
| --- | --- | --- | --- | --- | --- | --- | --- | --- | --- |
| 1107M | 1108M Group | 0 | 0 | 0 | 1 | 1 | 1 | 1 | 4 |
| 1108M | 1108M Group | 0 | 0 | 1 | 1 | 1 | 1 | 0 | 4 |
| 1106M | 8 Mile | 1 | 1 | 1 | 1 | 0 | 1 | 0 | 5 |
| 1154F | 8 Mile | 0 | 1 | 1 | 1 | 0 | 1 | 0 | 4 |
| 1231M | 8 Mile | 1 | 1 | 1 | 1 | 1 | 1 | 0 | 6 |
| 1232M | 8 Mile | 1 | 1 | 1 | 1 | 1 | 1 | 0 | 6 |
| 1233M | 8 Mile | 1 | 1 | 1 | 1 | 1 | 1 | 0 | 6 |
| 1049F | Alone | 0 | 0 | 1 | 1 | 1 | 1 | 0 | 4 |
| 1117M | Cougar Creek | 0 | 0 | 0 | 0 | 0 | 1 | 0 | 1 |
| 953F_1204F | Cougar Creek | 0 | 1 | 1 | 1 | 1 | 1 | 0 | 5 |
| 1047M | Junction Butte | 1 | 1 | 1 | 1 | 1 | 1 | 0 | 6 |
| 1048M | Junction Butte | 1 | 1 | 1 | 1 | 1 | 1 | 0 | 6 |
| 1109F | Junction Butte | 1 | 1 | 1 | 1 | 0 | 1 | 0 | 5 |
| 1229F | Junction Butte | 1 | 1 | 1 | 1 | 1 | 1 | 0 | 6 |
| 907F | Junction Butte | 1 | 1 | 1 | 1 | 1 | 1 | 0 | 6 |
| 969F | Junction Butte | 1 | 1 | 0 | 1 | 0 | 1 | 0 | 4 |
| 1014M | Wapiti Lake | 0 | 0 | 1 | 1 | 1 | 0 | 0 | 3 |
| 1104F | Wapiti Lake | 0 | 0 | 0 | 0 | 1 | 1 | 1 | 3 |
| 1105M | Wapiti Lake | 1 | 1 | 1 | 1 | 1 | 1 | 0 | 6 |
| 1155M | Wapiti Lake | 0 | 0 | 0 | 1 | 0 | 0 | 0 | 1 |
| 1203F | Wapiti Lake | 0 | 1 | 1 | 1 | 1 | 1 | 0 | 5 |
| 1234M | Wapiti Lake | 1 | 1 | 1 | 1 | 1 | 1 | 0 | 6 |
| 1235F | Wapiti Lake | 1 | 1 | 1 | 1 | 1 | 1 | 0 | 6 |
| 1236M | Wapiti Lake | 0 | 1 | 1 | 1 | 1 | 1 | 0 | 5 |
| ***TOTAL*** | - | 13 | 17 | 19 | 22 | 18 | 22 | 2 | 113 |

**Figure S4**. (a) PC1 (20.850%) and PC2 (15.840%) and (b) PC2 (15.840%) and PC3 (7.309%) calculated using unweighted UniFrac distances. Asterisks indicate heavily and moderately haired body sites.

**Figure S5**. Hierarchical clustering of body sites using Euclidean distances.

**Table S5**. Pairwise results from alpha diversity testing (observed ASVs) performed between body sites. Sample size is given in parentheses, and test statistics include Kruskal-Wallis *H* and Benjamini-Hochburg corrected *q*-values to account for multiple testing.

|  | ***Flank (17)*** | | ***Ear Canal (19)*** | | ***Feces (2)*** | | ***Lip (22)*** | | ***Nostril (18)*** | | ***Anus (22)*** | |
| --- | --- | --- | --- | --- | --- | --- | --- | --- | --- | --- | --- | --- |
|  | ***H*** | ***q-value*** | ***H*** | ***q-value*** | ***H*** | ***q-value*** | ***H*** | ***q-value*** | ***H*** | ***q-value*** | ***H*** | ***q-value*** |
| ***Axilla (13)*** | 0.369 | 0.571 | 0.106 | 0.744 | 2.890 | 0.151 | 2.469 | 0.163 | 10.010 | 0.006* | 6.925 | 0.022* |
| ***Flank (17)*** | . | . | 0.934 | 0.389 | 3.465 | 0.128 | 7.553 | 0.018* | 15.052 | 0.002* | 11.761 | 0.003* |
| ***Ear Canal (19)*** | . | . | . | . | 2.813 | 0.151 | 0.481 | 0.540 | 13.974 | 0.002* | 2.506 | 0.163 |
| ***Feces (2)*** | . | . | . | . | . | . | 3.350 | 0.128 | 1.018 | 0.387 | 1.853 | 0.228 |
| ***Lip (22)*** | . | . | . | . | . | . | . | . | 11.651 | 0.003* | 4.082 | 0.101 |
| ***Nostril (18)*** | . | . | . | . | . | . | . | . | . | . | 9.959 | 0.006* |

**Table S6.** Pairwise results from alpha diversity testing (Pielou’s evenness) performed between body sites. Sample size is given in parentheses, and test statistics include Kruskal-Wallis *H* and Benjamini-Hochburg corrected *q*-values to account for multiple testing.

|  | ***Flank (17)*** | | ***Ear Canal (19)*** | | ***Feces (2)*** | | ***Lip (22)*** | | ***Nostril (18)*** | | ***Perianal (22)*** | |
| --- | --- | --- | --- | --- | --- | --- | --- | --- | --- | --- | --- | --- |
|  | ***H*** | ***q-value*** | ***H*** | ***q-value*** | ***H*** | ***q-value*** | ***H*** | ***q-value*** | ***H*** | ***q-value*** | ***H*** | ***q-value*** |
| ***Axilla (13)*** | 1.325 | 0.309 | 2.182 | 0.209 | 2.337 | 0.204 | 10.741 | 0.004* | 6.771 | 0.020* | 15.683 | 0.001* |
| ***Flank (17)*** | . | . | 0.012 | 0.917 | 0.865 | 0.411 | 4.880 | 0.052 | 10.039 | 0.005* | 9.184 | 0.006* |
| ***Ear Canal (19)*** | . | . | . | . | 2.067 | 0.211 | 7.252 | 0.017* | 12.425 | 0.002* | 13.397 | 0.001* |
| ***Feces (2)*** | . | . | . | . | . | . | 0.011 | 0.917 | 4.063 | 0.077 | 1.571 | 0.276 |
| ***Lip (22)*** | . | . | . | . | . | . | . | . | 20.860 | <0.001* | 0.754 | 0.426 |
| ***Nostril (18)*** | . | . | . | . | . | . | . | . | . | . | 24.482 | <0.001* |

**Figure S6.** Taxonomic composition of each body site at the genus level, with taxa identifiers provided for the first 50 taxa (top down).

**Table S7.** Results from single-factor *PERMANOVA* implemented with the *diversity adonis* function in *QIIME 2* for perianal swabs (PA; proxy for gut microbial communities) and dorsal flank swabs (DF; proxy for skin microbial communities). Columns include variable and degrees of freedom (*df*), sum of squares (SS), mean squares (MS), test statistic (*F*), coefficient of determination *(R^2^*), and the significance (*p*) value. Variables are listed in order of highest R^2^ to lowest R^2^ within each beta diversity measure.

| **PA** | **Variable (*df)*** | ***SS*** | ***MS*** | ***F*** | ***R^2^*** | ***p*** |
| --- | --- | --- | --- | --- | --- | --- |
| Bray-Curtis | Sampling Pack (5) | 1.676 | 0.335 | 2.347 | 0.423 | 0.001* |
|  | Body Condition (4) | 1.204 | 0.301 | 1.856 | 0.304 | 0.008* |
|  | Field Season (2) | 0.519 | 0.260 | 1.434 | 0.131 | 0.113 |
|  | Age Class (2) | 0.355 | 0.178 | 0.936 | 0.090 | 0.511 |
|  | Sex (1) | 0.190 | 0.190 | 1.008 | 0.048 | 0.380 |
|  | Coat Color (1) | 0.150 | 0.150 | 0.788 | 0.038 | 0.614 |
| Unweighted UniFrac | Sampling Pack (5) | 0.505 | 0.101 | 1.488 | 0.317 | 0.015* |
|  | Body Condition (4) | 0.336 | 0.084 | 1.139 | 0.211 | 0.257 |
|  | Field Season (2) | 0.244 | 0.122 | 1.726 | 0.154 | 0.018* |
|  | Age Class (2) | 0.182 | 0.091 | 1.228 | 0.114 | 0.218 |
|  | Coat Color (1) | 0.091 | 0.091 | 1.210 | 0.057 | 0.255 |
|  | Sex (1) | 0.057 | 0.057 | 0.744 | 0.036 | 0.747 |
|  |  |  |  |  |  |  |
| **DF** | **Variable (*df)*** | ***SS*** | ***MS*** | ***F*** | ***R^2^*** | ***p*** |
| Bray-Curtis | Sampling Pack (3) | 1.405 | 0.468 | 1.138 | 0.208 | 0.088 |
|  | Field Season (2) | 0.970 | 0.485 | 1.175 | 0.144 | 0.070 |
|  | Age Class (2) | 0.962 | 0.481 | 1.162 | 0.142 | 0.088 |
|  | Body Condition (2) | 0.865 | 0.432 | 1.028 | 0.128 | 0.387 |
|  | Sex (1) | 0.476 | 0.476 | 1.139 | 0.071 | 0.154 |
|  | Coat Color (1) | 0.449 | 0.449 | 1.068 | 0.066 | 0.264 |
| Unweighted UniFrac | Sampling Pack (3) | 0.639 | 0.213 | 1.490 | 0.256 | 0.007* |
|  | Field Season (2) | 0.505 | 0.252 | 1.775 | 0.202 | 0.001* |
|  | Body Condition (2) | 0.386 | 0.193 | 1.280 | 0.155 | 0.075 |
|  | Age Class (2) | 0.333 | 0.167 | 1.079 | 0.134 | 0.309 |
|  | Coat Color (1) | 0.192 | 0.192 | 1.251 | 0.077 | 0.145 |
|  | Sex (1) | 0.189 | 0.189 | 1.226 | 0.076 | 0.133 |

**Table S8.** Results from multi-factor *PERMANOVA* implemented with the *diversity adonis* function in *QIIME 2* for perianal swabs (PA; proxy for gut microbial communities) and dorsal flank swabs (DF; proxy for skin microbial communities). Columns include variable and degrees of freedom (*df*), sum of squares (SS), mean squares (MS), test statistic (*F*), coefficient of determination *(R^2^*), and the significance (*p*) value. Variables are listed in the order they were input into the model (based on descending values of R^2^ for single-factor tests).

| **PA** | **Variable (*df)*** | ***SS*** | ***MS*** | ***F*** | ***R^2^*** | ***p*** |
| --- | --- | --- | --- | --- | --- | --- |
| Bray-Curtis | SamplingPack (5) | 1.676 | 0.335 | 2.142 | 0.423 | 0.004* |
|  | BodyCondition (4) | 0.527 | 0.132 | 0.841 | 0.133 | 0.681 |
|  | FieldSeason (2) | 0.364 | 0.182 | 1.163 | 0.092 | 0.248 |
|  | AgeClass (2) | 0.296 | 0.148 | 0.945 | 0.075 | 0.501 |
|  | Sex (1) | 0.050 | 0.050 | 0.320 | 0.013 | 0.983 |
|  | CoatColor (1) | 0.110 | 0.110 | 0.701 | 0.028 | 0.738 |
|  | Residuals (6) | 0.939 | 0.157 | NA | 0.237 | NA |
|  | Total (21) | 3.960 | NA | NA | 1 | NA |
| Unweighted UniFrac | SamplingPack (5) | 0.505 | 0.101 | 1.472 | 0.317 | 0.030* |
|  | BodyCondition (4) | 0.273 | 0.068 | 0.997 | 0.172 | 0.524 |
|  | FieldSeason (2) | 0.187 | 0.093 | 1.362 | 0.118 | 0.148 |
|  | AgeClass (2) | 0.121 | 0.061 | 0.885 | 0.076 | 0.641 |
|  | CoatColor (1) | 0.050 | 0.050 | 0.729 | 0.031 | 0.756 |
|  | Sex (1) | 0.042 | 0.042 | 0.612 | 0.026 | 0.856 |
|  | Residuals (6) | 0.412 | 0.069 | NA | 0.259 | NA |
|  | Total (21) | 1.590 | NA | NA | 1 | NA |
|  |  |  |  |  |  |  |
| **DF** | **Variable (*df)*** | ***SS*** | ***MS*** | ***F*** | ***R^2^*** | ***p*** |
| Bray-Curtis | SamplingPack (3) | 1.405 | 0.468 | 1.219 | 0.208 | 0.039* |
|  | FieldSeason (2) | 0.941 | 0.471 | 1.225 | 0.139 | 0.054 |
|  | AgeClass (2) | 0.780 | 0.390 | 1.015 | 0.116 | 0.439 |
|  | BodyCondition (2) | 0.832 | 0.416 | 1.083 | 0.123 | 0.224 |
|  | Sex (1) | 0.389 | 0.389 | 1.013 | 0.058 | 0.508 |
|  | CoatColor (1) | 0.484 | 0.484 | 1.260 | 0.072 | 0.046* |
|  | Residuals (5) | 1.921 | 0.384 | NA | 0.284 | NA |
|  | Total (16) | 6.752 | NA | NA | 1.000 | NA |
| Unweighted UniFrac | SamplingPack (3) | 0.639 | 0.213 | 1.728 | 0.256 | 0.002* |
|  | FieldSeason (2) | 0.488 | 0.244 | 1.980 | 0.195 | 0.002* |
|  | BodyCondition (2) | 0.223 | 0.111 | 0.904 | 0.089 | 0.668 |
|  | AgeClass (2) | 0.218 | 0.109 | 0.883 | 0.087 | 0.718 |
|  | CoatColor (1) | 0.157 | 0.157 | 1.277 | 0.063 | 0.139 |
|  | Sex (1) | 0.156 | 0.156 | 1.265 | 0.062 | 0.181 |
|  | Residuals (5) | 0.616 | 0.123 | NA | 0.247 | NA |
|  | Total (16) | 2.496 | NA | NA | 1.000 | NA |

**Figure S7.** Bray-Curtis PCoA plots for (a) perianal area and (b) dorsal flank swabs colored by sampling pack. Unweighted UniFrac PCoA plots for (c) perianal area and (d) dorsal flank swabs colored by sampling pack.

**Table S9.** Results obtained from full and partial Mantel tests assessing correlation between genetic and microbial distances. Genetic distances were calculated using the Euclidean distance metric with two SNP datasets (517 SNPs and 86,545 SNPs). Microbial distances were calculated using the Bray-Curtis dissimilarity index to measure species abundance and unweighted UniFrac distances to measure species presence in perianal (PA) and dorsal flank (DF) samples. Partial Mantel tests were implemented with a third distance matrix for pack membership, where 0=same pack and 1=different pack. Asterisks indicate statistically significant results.

|  |  |  | ***full Mantel test*** | | ***partial Mantel test*** | |
| --- | --- | --- | --- | --- | --- | --- |
|  | **Genetic Distance (SNPs)** | **Microbiome Distance** | ***r-*value** | ***p-*value** | ***r-*value** | ***p-*value** |
| **PA** | Euclidean (517) | Bray-Curtis | 0.245 | 0.004* | 0.190 | 0.010* |
|  | Euclidean (517) | Unweighted UniFrac | 0.168 | 0.018* | 0.120 | 0.052 |
|  | Euclidean (86,545) | Bray-Curtis | 0.411 | 0.001* | 0.376 | 0.001* |
|  | Euclidean (86,545) | Unweighted UniFrac | 0.245 | 0.005* | 0.206 | 0.019* |
| **DF** | Euclidean (517) | Bray-Curtis | 0.204 | 0.062 | 0.168 | 0.108 |
|  | Euclidean (517) | Unweighted UniFrac | 0.253 | 0.026* | 0.172 | 0.091 |
|  | Euclidean (86,545) | Bray-Curtis | 0.091 | 0.285 | 0.039 | 0.376 |
|  | Euclidean (86,545) | Unweighted UniFrac | 0.092 | 0.231 | -0.015 | 0.520 |
